# Sparse labels, no problems: Infant categorization under challenging conditions

**DOI:** 10.1111/cdev.13818

**Published:** 2022-06-22

**Authors:** Alexander LaTourrette, Sandra R. Waxman

**Affiliations:** ^1^ University of Pennsylvania Philadelphia Pennsylvania USA; ^2^ Northwestern University Evanston Illinois USA

## Abstract

Labeling promotes infants' object categorization even when labels are rare. By 2 years, infants engage in “semi‐supervised learning” (SSL), integrating labeled and unlabeled exemplars to learn categories. However, everyday learning contexts pose substantial challenges for infants' SSL. Here, two studies (*n* = 74, 51% female, 62% non‐Hispanic White, 18% multiracial, 8% Asian, 6% Black, *M*_age_ = 27.3 months, collected 2018–2020) implemented a familiarization‐novelty preference paradigm assessing 2‐year‐olds' SSL when (i) exemplars from the target category are interspersed with other objects (Study 1, *d* = .67) and (ii) multiple categories are learned simultaneously (Study 2, *d* = .74). The findings indicate 2‐year‐olds' SSL is robust enough to support object categorization despite substantial challenges posed by everyday learning contexts.

**Abbreviation**
SSL: semi‐supervised learning

Infants learn object categories from messy, variable, and often sparse input. An infant might first encounter tulips on a walk in early spring—a clear opportunity for category learning. If the infant's caregiver is engaged with the infant and botanically inclined, the infant might even hear a few of these new objects labeled with the same noun, “tulip.” Nevertheless, most of the tulips the infant sees will likely go unlabeled. Furthermore, these tulips will be interspersed with labeled and unlabeled members of other categories: perhaps the infant will encounter newly blooming daffodils as well, in addition to various bushes, bees, people, cars, and the rest of the everyday world's “blooming, buzzing confusion” (James, 1890).

Infants' caregivers, and the object labels they provide, offer infants a powerful resource for category learning. Substantial research has shown that from early infancy, object labeling supports categorization. Moreover, infants are sensitive not just to *whether* objects are labeled but to how those labels are applied. Infants as young as 10 to 14 months form an object category more successfully when members of that category are given with a consistent label (e.g., “Look, a tulip!”) than when the objects remain unlabeled or when each is given a distinct label (Balaban & Waxman, 1996; Plunkett et al., 2008; Waxman & Braun, 2005; Waxman & Markow, 1995). In contrast, providing distinct names for distinct individual objects facilitates infants' ability to represent the objects as individuals (LaTourrette & Waxman, 2020; Pickron et al., 2018; Scott, 2011; Scott & Monesson, 2010; Xu & Carey, 1996).

Most of the evidence documenting these effects of labels on early category learning relies on experimental paradigms in which each member of the object category is labeled. Notice, however, that this stands in stark contrast to infants' daily lives, where even the most attentive caregiver cannot label every new object (or even every tulip) as their child sees it. Indeed, there is substantial variation both within and across cultures in the frequency with which objects are labeled (e.g., Cartmill et al., 2013; Gaskins, 1999; Lieven, 1994; Rogoff et al., 1993; Shneidman & Goldin‐Meadow, 2012). Furthermore, even when caregivers do provide a label, its intended referent may well be unclear from the visual context alone (Cartmill et al., 2013; Gillette et al., 1999; Medina et al., 2011; Piccin & Waxman, 2007). How, then, can we reconcile the scarcity of labeling in infants' everyday environments with the power of labeling in infants' category learning?

One potential solution is for learners to draw on both labeled and unlabeled exemplars—using labeled exemplars to estimate categories which are then fleshed out by unlabeled exemplars. Indeed, this approach is already extensively employed in the field of machine learning. Semi‐supervised learning (SSL) algorithms' integration of labeled and unlabeled data provides an efficient way of solving machine learning problems featuring sparsely labeled datasets, including object classification and person identification (Balcan et al., 2005; Board & Pitt, 1989; Chapelle et al., 2006; Cheplygina et al., 2019; Lu et al., 2013; Xu et al., 2009; Zhu & Goldberg, 2009). Algorithmic approaches vary (for a review, see van Engelen & Hoos, 2020; for a review of earlier, related approaches, see Vapnik, 2006), but the simplest approaches, including “wrapper” algorithms, typically take advantage of labeled exemplars to create initial estimates of category boundaries. The algorithms then use these initial, labeled exemplars to generate hypotheses about the category membership of subsequent unlabeled exemplars and incorporate these new exemplars into their hypothesized categories, with varying levels of confidence. In this way, SSL integrates initial, labeled exemplars and subsequent, unlabeled exemplars to learn categories. Moreover, evidence suggests that SSL supports object categorization in both human adults (e.g., Kalish et al., 2011; Lake & McClelland, 2011; Patterson & Kurtz, 2018; Zhu et al., 2007; though see Vandist et al., 2009) and children (Kalish et al., 2015). Indeed, SSL may be especially useful when learning ambiguous categories for which the relevant dimensions are salient to the learner (Bröker et al., 2022; Vong et al., 2015). In such contexts, both adults and children appear to use labeled exemplars to generate initial category representations which are then modified to fit the unlabeled exemplars (for a review, see Gibson et al., 2013).

Recent evidence has also demonstrated semi‐supervised category learning in children as young as 2 years of age. LaTourrette and Waxman (2019) developed a novelty preference task to test 2‐year‐olds' ability to learn a single category in a sparse labeling context. Infants learned the object category successfully in a Fully Supervised condition (all six exemplars labeled with the same name) but not an Unsupervised condition (no exemplars labeled). More critically, infants also successfully learned the category in a Semi‐supervised condition, in which only the first two of six exemplars were labeled. Infants in this condition categorized as successfully as those in the Fully Supervised condition. Importantly, the timing of the labeling mattered: infants in a Reversed SSL condition, in which the *final* two exemplars were labeled, failed to form object categories. This suggests that providing two named exemplars was not, on its own, sufficient for successful learning and that infants did not integrate previously seen unlabeled exemplars with the labeled exemplars. Thus, infants' success in the Semi‐supervised condition indicates they engaged in online SSL: learning the category by integrating labeled exemplars with subsequent, unlabeled exemplars.

These findings provide a strong proof‐of‐concept: infants as young as 2 years of age are capable of SSL. Indeed, because most of the object categories infants encounter are likely to be sparsely labeled for them, SSL may play a powerful and ubiquitous role in infants' daily lives.

However, it remains unknown whether SSL is robust to the more difficult challenges infants face in everyday learning environments. After all, like the infant observing tulips on a springtime walk, infants typically encounter intermixed members of different object categories—not just members of a single category, as in LaTourrette & Waxman's design. Indeed, Clerkin et al. (2017) estimated that infants' visual perspectives in at‐home contexts contain an average of nearly eight distinct kinds of objects at any given moment. Rather than appearing *en masse* and with no interruptions, category exemplars are typically interspersed with members of other categories.

Here, our goal is to assess whether 2‐year‐olds' SSL abilities can support category learning in these more varied environments. To succeed, infants must identify the relevant candidate exemplars (e.g., including tulips, but excluding daffodils, bushes, and bees) and integrate them into an emerging object category (tulips). If infants successfully do this, then their performance in such environments should resemble that of the SSL condition in LaTourrette and Waxman's (2019) single‐category learning task. If infants cannot do so, their categorization should be disrupted in contexts containing multiple, unrelated objects, as infants are unable to identify the relevant, unlabeled exemplars. That is, when confronted with unlabeled objects which clearly do not belong to the recently labeled category, perhaps infants infer that *none* of the unlabeled objects belong to that category—a substantial limitation on SSL. A second critical challenge is that infants are typically exposed to members of new object categories mixed together (e.g., learning about both tulips and daffodils). To acquire multiple categories simultaneously, infants must accurately decide which of their emerging categories to incorporate each new exemplar into.

We address each of these challenges in turn, modifying LaTourrette and Waxman's (2019) paradigm to test infants' category learning under more realistic constraints. In Study 1, we ask whether 2‐year‐olds use SSL to successfully form object categories in varied contexts, in which unlabeled members of the target category are interspersed with distractor objects from different categories. In Study 2, we introduce an even more difficult challenge, asking whether 2‐year‐olds' SSL is sufficiently robust to form two novel categories simultaneously. In particular, if infants use online SSL to learn multiple categories simultaneously, they should succeed only when given the opportunity to integrate initial, labeled exemplars with subsequent unlabeled exemplars—and fail when labeled exemplars are presented at the end of learning. Together, these experiments assess the robustness of SSL in infancy and its viability as a mechanism for everyday category learning.

## STUDY 1: VARIED CONTEXTS

First, we ask whether 2‐year‐olds can learn a new category under sparse labeling when the learning phase includes not only unnamed objects from the target category but objects from other categories (i.e., “distractors”) as well. If infants' SSL is robust enough to facilitate everyday learning, then infants should successfully form categories under these conditions. If infants fail to do so, then SSL may play a more limited role in category learning outside the laboratory.

### Method

#### Participants

Twenty‐four infants between 24 and 30 months (*M* = 27.3, *SD* = 1.39; 11 females, 13 males; 19 White, 5 multiracial) were recruited from predominantly college‐educated families in 2018. This sample size yields 85% power based on LaTourrette and Waxman's (2019) previous effect size for a two‐tailed *t*‐test, *d* = .63. To facilitate direct comparisons with LaTourrette and Waxman (2019), we imposed their same inclusion criteria, for both Studies 1 and 2. More specifically, to be included in analysis, we required that infants accumulate 25% looking to at least four of six target exemplars (2 infants in Study 1 were excluded for failing to meet this criterion) and at least 2500 ms of looking at test (1 infant excluded). Another five infants were excluded for technical errors. Finally, following LaTourrette & Waxman, we eliminated outliers with the interquartile range rule (0 infants excluded).

#### Apparatus

A Tobii T60XL eye‐tracker was used for stimulus presentation and data collection. The eye‐tracker has a sampling rate of 60 Hz and a display size of 57.3 × 45 cm.

#### Stimuli

All target stimuli were identical to LaTourrette and Waxman (2019). Auditory stimuli consisted of labeling phrases (e.g., “Look at the modi!”) and non‐labeling phrases (e.g., “Look at that!”) produced by a female, native English speaker using infant‐directed speech and recorded in a sound isolation booth.

**FIGURE 1:**
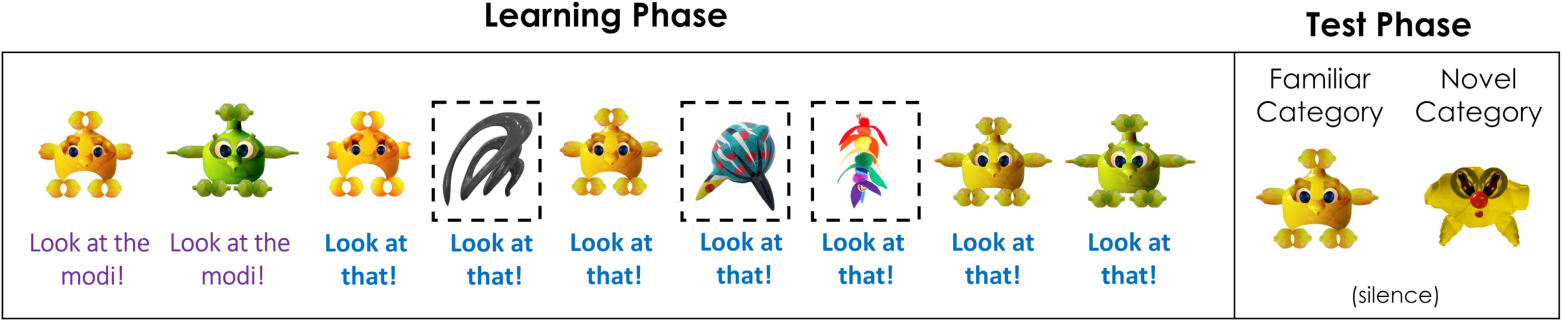
Design for Study 1. A representative set of stimuli. Two‐year‐olds viewed a series of nine exemplars during the Learning Phase: six from the target category and three distractors. Dashed boxes indicate distractor exemplars. Only the first two objects were labeled. At Test, infants viewed a familiar and novel category member, presented simultaneously in silence.

Visual stimuli included members of two novel categories, created by Havy and Waxman (2016) and downloaded from https://osf.io/n6uy8/ (see Figure 1). Each category represents a continuous spectrum created by morphing two colorful animate creatures to create a continuum of exemplars that vary along multiple dimensions (e.g., color, shape, body proportions, and individual feature details). The six category exemplars presented during learning were selected at equal intervals from the continua: the most extreme exemplar from each end of the continuum as well as the 20%, 40%, 60%, and 80% exemplars on the continuum. Each infant viewed only one category.

In addition, infants also viewed 3 novel, colorful artifacts as distractors (see Figure 1). These inanimate artifact distractors differed considerably from one another and even more so from members of the target categories, which were all animate individuals with eyes and faces. Prior evidence indicates that 2‐year‐olds are unlikely to consider such distractors to be members of the animate target categories (Anderson et al., 2018; Rakison & Poulin‐Dubois, 2001; Spriet et al., 2021; Surian & Caldi, 2010).

At test, infants viewed new exemplars from the now‐familiar category (seen during learning) and from an unfamiliar novel category (never seen before). For the familiar category exemplar, we used the midpoint of the familiar category's continuum, ensuring infants were tested on a representative category member not seen during learning. For the novel category exemplar, we selected the midpoint exemplar from one of two other continuous categories and matched it in color to the respective familiar category test exemplar (see Figure 1).

#### Procedure

The procedure was identical to LaTourrette and Waxman's (2019) Semi‐supervised condition with one exception: in addition to the target category exemplars, infants were also shown unlabeled *distractors*. As a result, in the Learning Phase, infants viewed a series of nine exemplars presented for 3 s each and paired with either a labeling phrase (first two exemplars, drawn from opposing sides of the category spectrum) or a non‐labeling phrase (other exemplars).

Distractor exemplars were intermixed with the unlabeled target category exemplars in one of two pseudorandom orders (see, e.g., Figure 1). The target category was counterbalanced across infants: each infant saw only one category. At test, infants viewed two new exemplars: the midpoint exemplar of the familiar category and the midpoint exemplar of a novel category, not seen before. These images were presented simultaneously and in silence for 10 s. If infants formed the category during learning, they should spend more time looking to the novel category exemplar than the familiar category exemplar; if infants failed to form the category, they should show no preference.

#### Data preparation

Following LaTourrette and Waxman (2019), we calculated two measures of infants' performance at test, focusing our analyses solely on infants' first 5 s of accumulated looking at test. All infants accumulated 5 s of looking at test. We then compared performance to LaTourrette and Waxman's Semi‐supervised condition, downloaded from a public repository at https://github.com/sandylat/ssl‐in‐infancy.

To calculate infants' *aggregate novelty preference*, we divided the time infants spent looking to the novel category exemplar by the total time spent looking to both exemplars. This proportion was then logit‐transformed for analysis with linear models. To evaluate the *gaze time course*, we used a cluster‐based permutations test (Maris & Oostenveld, 2007), comparing infants' looking preference in each gaze sample (equivalent to a 16.67 ms time‐bin) across the conditions using a binomial logistic regression with an alpha level of .05. For adjacent samples that yielded significant differences, we summed together the *t*‐statistics (calculated as coefficient divided by standard error) to create a cumulative *t*‐statistic representing the total size of the divergence between conditions in that window. To evaluate the probability of such a divergence occurring by chance, we conducted 1000 simulations with randomly shuffled condition labels and compared the target divergence(s) against this chance‐based distribution, thus controlling for Type 1 error.

### Results

#### Learning phase

Infants' average attention to the six target exemplars (*M* = 2.19 s per exemplar, *SD* = 0.41) did not differ from their attention to the distractor exemplars (*M* = 2.29 s, *SD* = 0.42), *t*(23) = 0.86, *p* = .40. Thus, infants were highly attentive to all learning exemplars.

#### Test phase

Preliminary analyses identified no effects of age, *p* = .95; sex, *p* = .72; or test position, *p* = .79. There was, however, an unanticipated effect of category. Although prior work with the same visual stimuli produced no category differences (Havy & Waxman, 2016; LaTourrette & Waxman, 2019), our preliminary analysis here revealed a significant effect of category, *t*(22) = 3.50, *p* = .002, *d* = .48, indicating novelty preferences were stronger for one category (*M* = 0.66, *SD* = 0.07) than the other (*M* = 0.52, *SD* = 0.11). Because this effect was unanticipated, and because conducting analyses for each category alone would be under‐powered, we proceeded with our analysis plan, using the mean of the two categories to test categorization. This analysis revealed a significant novelty preference (*M* = 0.58, *SD* = 0.12), *t*(23) = 3.33, *p* = .0029, *d* = .67, indicating successful categorization.

**FIGURE 2:**
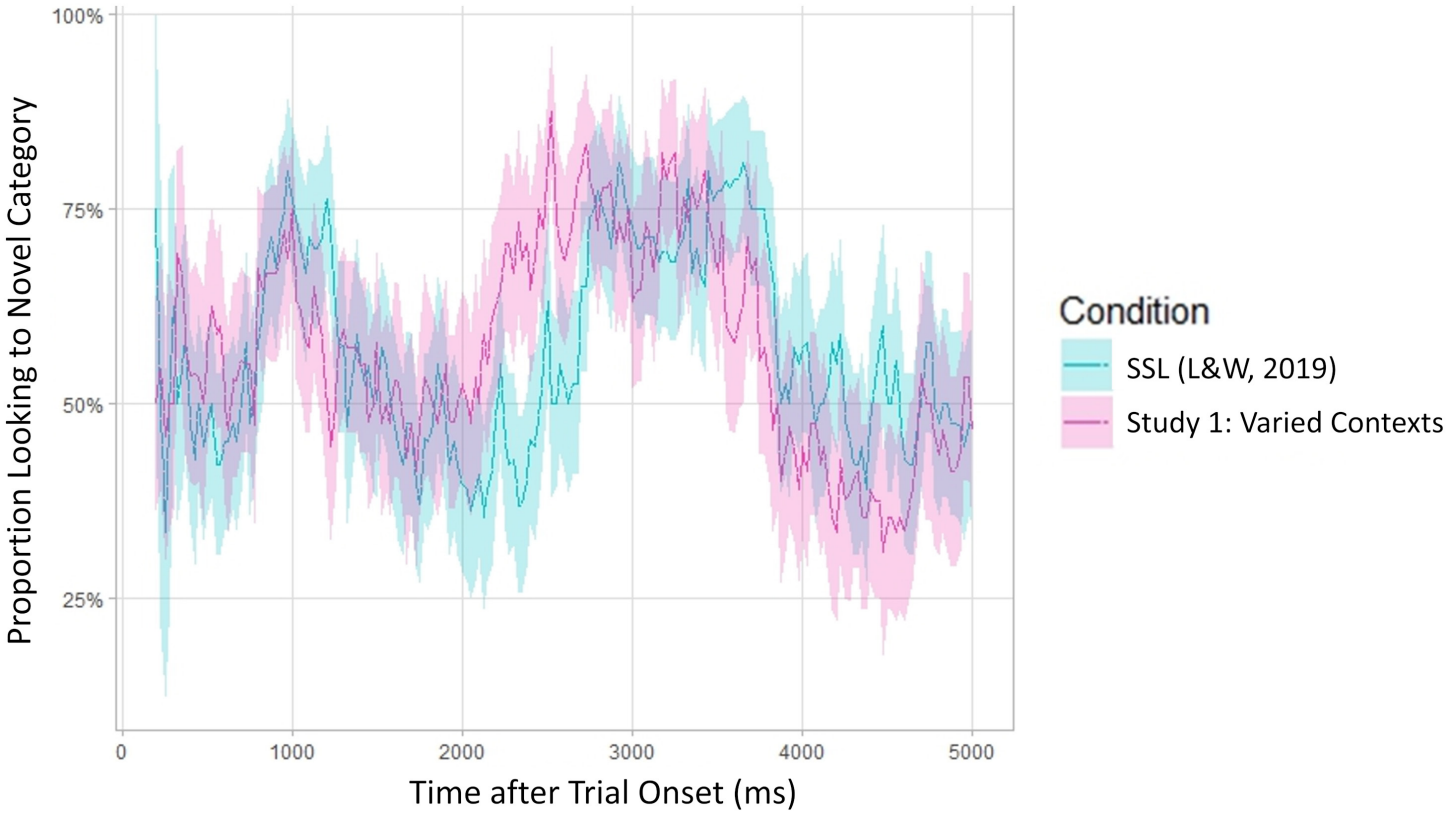
Study 1 gaze time course. Performance in Study 1 did not significantly diverge from LaTourrette and Waxman's (2019) SSL condition, *p* > .6. Shaded regions indicate *SEM*.

Moreover, infants' performance did not differ from performance in LaTourrette and Waxman's (2019) Semi‐supervised condition (*M* = 0.59, *SD* = 0.14) on either aggregate looking preference, *t*(46) = 0.31, *p* = .76, *d* = .09, or in the cluster‐based permutations test, *p* > .6 (see Figure 2). Thus, adding distractor objects from unrelated categories posed no discernible difficulty for infants' SSL capacities.

### Discussion

Even in a sparsely labeled environment featuring objects from different categories, 2‐year‐olds' SSL was sufficiently robust to facilitate learning of the target category. This outcome is encouraging for the viability of SSL in everyday contexts, but it also raises a new challenge: can infants use SSL to go one step further? Can infants acquire two new categories simultaneously, by selectively identifying and incorporating labeled and unlabeled exemplars of each?

## STUDY 2: LEARNING MULTIPLE CATEGORIES

We therefore tested whether 2‐year‐olds' SSL is sufficiently robust to support the acquisition of two distinct object categories in sparsely labeled environments. Our design builds upon evidence demonstrating that labels support infants' ability to form two new categories simultaneously. When infants are presented with exemplars from two coherent object categories, and *every* exemplar is labeled accordingly, infants successfully learn both categories (e.g., Althaus & Westermann, 2016; Havy & Waxman, 2016; Plunkett et al., 2008). However, it remains unknown whether they succeed in such a task when only some of the exemplars are labeled. To succeed, infants must initiate two distinct category representations and then identify which of the subsequent, unlabeled exemplars should be incorporated into each category.

To assess this capacity, we compared infants' performance in two sparse labeling conditions, differing only in when labeled exemplars were introduced. In the Simultaneous Semi‐supervised condition, infants first viewed two labeled exemplars from each of the two categories, followed by four unlabeled exemplars from each, intermixed. In the Reversed Control condition, infants viewed the same exemplars, but the labeled exemplars were presented at the end of learning. If infants engage in online SSL, then they should succeed only in the Simultaneous Semi‐supervised condition, where the labeled exemplars can initiate the formation of two distinct categories that are then refined by unlabeled exemplars.

### Method

#### Participants

Fifty infants between 25 and 30 months of age (*M* = 27.3, *SD* = 1.39; 27 females, 23 males; 27 White, 8 multiracial, 6 Asian, 4 Black, 1 American Indian, 1 Hispanic or Latinx, and 3 unreported) were recruited in Spring 2019–Winter 2020 from predominantly college‐educated families with demographic diversity representative of the Evanston, IL area. An additional 13 participants were excluded for technical errors (3), failing to accumulate 25% looking to at least four of six exemplars for both categories (6), or failing to accumulate at least 2500 ms of looking on both test trials (4). Two test trials (one in each condition) were excluded as outlier scores and replaced by running an additional participant, yielding 24 test trials per category in each condition.

#### Procedure

**FIGURE 3:**
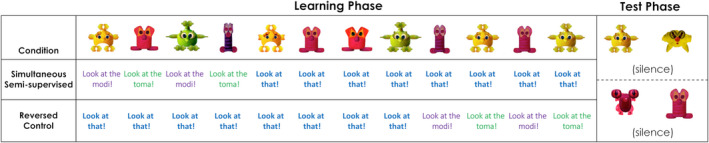
Design for Study 2. During learning, infants viewed exemplars from two categories (2 labeled and 4 unlabeled exemplars from each). The order of the labeled and unlabeled exemplars varied by condition. Next, infants proceeded to categorization tests for Category 1 (upper) and Category 2 (lower), presented in counterbalanced order.

Infants were randomly assigned to either the Simultaneous Semi‐supervised condition or the Reversed Control condition. The exemplars infants viewed were the same as in Study 1 (six exemplars from each of two novel categories), but each infant now viewed both categories (Figure 3). Categories were presented in an ABABABBABABA order, counterbalancing the categories. In the Simultaneous Semi‐supervised condition, the first four exemplars were all labeled for infants (e.g., “Look at the modi/toma!”) and the final eight exemplars were paired with non‐labeling phrases (e.g., “Look at that!”). In the Reversed Control condition, the four labeled exemplars were moved to the end of the learning phase, which now began with the eight unlabeled exemplars. In both conditions, exemplars were shown in one of four pseudorandom orders constructed such that each category's labeled exemplars came from opposite sides of the continuum (e.g., the 80% and 40% exemplars).

Test trials were identical to Study 1. Infants saw test trials for both categories, counterbalancing the order and the novel category exemplar's left/right position. We analyzed the first 5 s of accumulated looking; infants contributed an average of 4.7 s of looking (*SD* = 0.38). Cluster‐based permutation tests were again performed over each sample, but with preferences averaged over the two categories' test trials and compared via *t*‐test.

### Results

#### Learning phase

Infants' attention to the exemplars in the Simultaneous Semi‐supervised (*M* = 2.26 s per exemplar, *SD* = 0.38) and Reversed Control (*M* = 2.31, *SD* = 0.38) conditions did not differ, *t*(48) = 0.42, *p* = .67, *d* = .12. Nor did attention to the labeled exemplars differ across Reversed Control (*M* = 2.41 s, *SD* = 0.37) and Simultaneous SSL conditions (*M* = 2.46 s, *SD* = 0.34), *t*(48) = 0.54, *p* = .59, *d* = .15.

#### Test phase

Preliminary analyses revealed no effects of age, *p* = .79, sex, *p* = .23, exemplar order, *p* = .56, test position, *p* = .17, or test trial order, *p* = .34. We submitted infants' novelty preference scores to a 2 (Condition) × 2 (Category) mixed effects ANOVA. As predicted, a main effect of condition, *F*(1, 40) = 4.77, *p* = .035, $\eta_{p}^{2}$ = .11, indicated that infants in the Simultaneous Semi‐supervised condition (*M* = 0.60, *SD* = 0.10) exhibited stronger novelty preferences than those in the Reversed Control (*M* = 0.52, *SD* = 0.11). This pattern indicates that infants successfully learned the two categories in the Simultaneous Semi‐supervised, but not Reversed Control, condition.

Additionally, we observed a marginal effect of category, *F*(1, 40) = 4.07, *p* = .051, $\eta_{p}^{2}$ = .09, but no significant interaction between condition and category, *F*(1, 40) = 2.09, *p* = .16, $\eta_{p}^{2}$ = .05. Critically, infants in the Simultaneous Semi‐supervised condition showed significant novelty preferences for *both* Category 1 (*M* = 0.55, *SD* = 0.11, *t*[24] = 2.34, *p* = .028, *d* = .47) and Category 2 (*M* = 0.64, *SD* = 0.13, *t*[24] = 5.02, *p* < .001, *d* = 1.00). In contrast, infants in the Reversed Control condition failed to show novelty preferences for either category (*M*_Category1_ = 0.51, *SD*_Category1_ = 0.13, *M*_Category2_ = 0.53, *SD*_Category2_ = 0.13), *p*s > .25, *d*s < .25.

**FIGURE 4:**
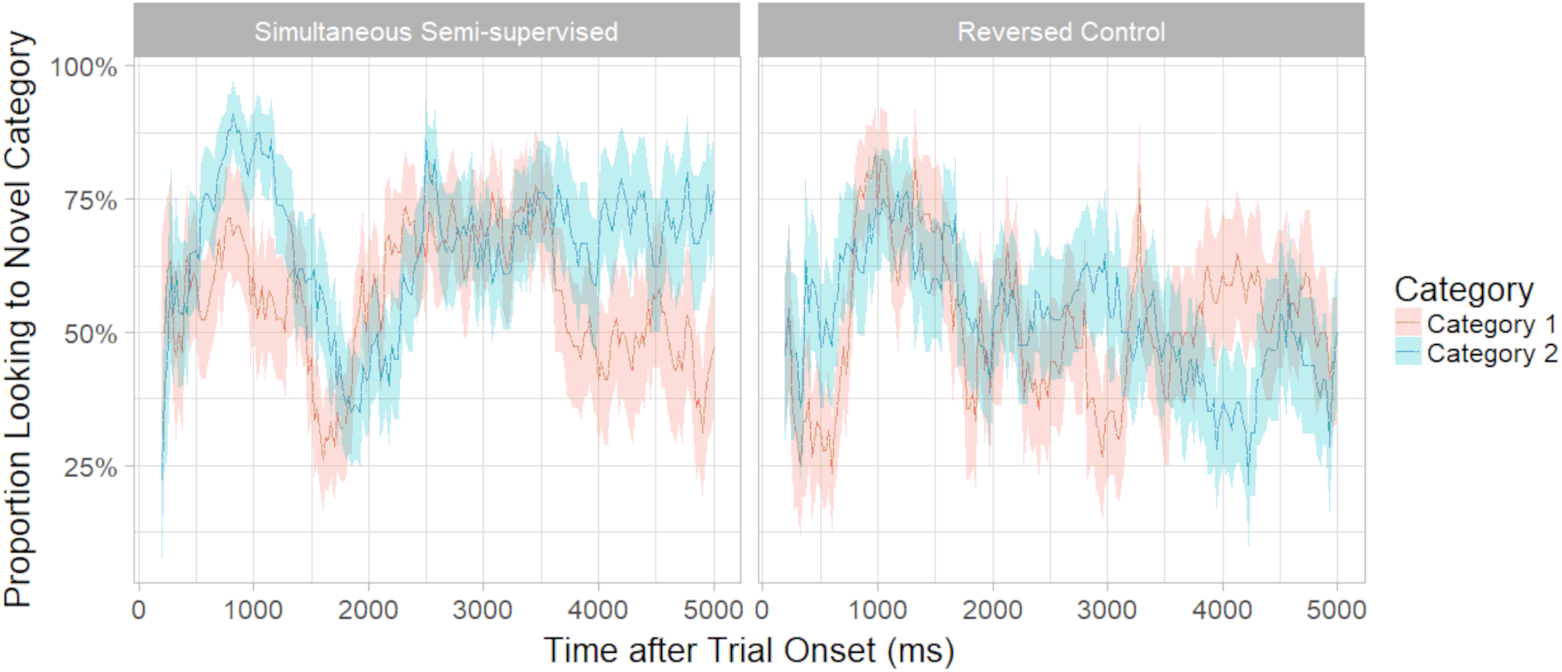
Study 2 gaze time course. Gaze patterns did not diverge by category in either the Simultaneous Semi‐supervised (at left) or Reversed Control (at right) conditions, nor did the conditions significantly diverge from each other, *p*s > .1. Shaded regions indicate *SEM*.

We then evaluated the gaze time course. To begin, we examined infants' performance by category: a cluster‐based permutations test found no significant divergences between looking patterns for Categories 1 and 2 within either the Simultaneous Semi‐supervised condition, *p*s > .6 or the Reversed Control condition, *p*s > .8 (see Figure 4). We, therefore, collapsed across categories to compare conditions. However, no significant divergences emerged between the conditions, *p*s > .1.

Finally, to determine whether infants in each of these conditions showed signs of successful learning in the time course, we compared each condition against LaTourrette & Waxman's Semi‐supervised and Unsupervised conditions. As predicted, infants' performance in the Simultaneous Semi‐supervised condition did not diverge from LaTourrette & Waxman's Semi‐supervised condition, *p* > .6, but did significantly diverge from the Unsupervised condition, showing a stronger novelty preference between 2383 and 3333 ms, cumulative *t* = 156.5, *p* = .001. In contrast, performance in the Reversed control condition essentially mirrored the Unsupervised condition, with no significant divergences, *p* > .75, though performance also did not diverge from the Semi‐supervised condition, *p* > .3. Thus, infants showed significant evidence of successful categorization only in the Simultaneous Semi‐supervised condition.

## GENERAL DISCUSSION

This work provides new insight into the power of infants' SSL to surmount some of the most daunting challenges posed by everyday category learning. Across experiments, infants benefited from hearing just two exemplars labeled—even when subsequent exemplars remained unlabeled. In Study 1, infants successfully employed SSL despite the presence of unlabeled distractor objects. In Study 2, infants used SSL to learn two distinct categories simultaneously. Moreover, infants succeeded only in the Simultaneous Semi‐supervised condition, not the Reversed Control: this suggests learning occurred online, with infants using labeled exemplars to initiate category formation and then incorporating subsequent, unlabeled exemplars into those categories. Thus, our tulip‐curious infant is likely to successfully learn the tulip category on their springtime walk, by integrating labeled and unlabeled tulip exemplars encountered amidst all the daffodils and sundry other objects.

These results reveal impressive developmental continuity. Infants successfully navigated one of the most difficult problems posed by SSL: accurately incorporating new unlabeled exemplars into their target categories. While this ability has been demonstrated in older children and adults (e.g., Gibson et al., 2013; Kalish et al., 2015; Lake & McClelland, 2011), the findings reported here provide the first demonstration of this ability in infancy.

These new findings provide confirmatory support for 2‐year‐olds' ability to engage in SSL, extending the results of LaTourrette and Waxman (2019), but more broadly, these studies remain exploratory in documenting the extent to which infants use SSL to acquire categories in more realistic and challenging environments. As such, they also raise many other possible lines of investigation regarding the interactions between labeled and unlabeled exemplars in infants' categorization. For instance, while we suggest infants' failure to learn categories in the Reversed Control condition stems from difficulty integrating labeled and unlabeled exemplars when labeled exemplars are encountered last, it is also possible that encountering a series of unlabeled exemplars could have instead impaired infants' ability to learn from the subsequent, labeled exemplars. Indeed, this interplay between semi‐supervised and unsupervised learning raises multiple questions, including how infants learn categories when labeled and unlabeled exemplars are interleaved, when labels are applied to only a particular subset of the target category (e.g., to pink but not white tulips), or when infants forget the category's label during learning. Future research might also test the generalizability of these findings to other object categories and to more socioeconomically or linguistically diverse infant populations. Finally, it will be important to discover whether infants can take advantage of SSL when the intended referent of a novel word remains unclear, when delays occur between exposures to category members, and when infants are acquiring hierarchically related categories (e.g., tulip and flower).

By considering the challenges that everyday learning contexts pose for infants, we can begin to identify the strategies that fuel their learning. Our findings demonstrate that in the sparsely labeled and varied contexts that characterize many everyday learning environments, infants successfully employ SSL to form new categories.

## CONFLICT OF INTEREST

None declared.
